# Online Wind Mapping for Coupled Path Planning and Contouring Control of Quadrotors

**DOI:** 10.3390/s26165250

**Published:** 2026-08-19

**Authors:** Mitchell Torok, Man Ching Melvin Chan, Donglin Sui, Mohammad Deghat

**Affiliations:** School of Mechanical and Manufacturing Engineering, University of New South Wales, Sydney, NSW 2052, Australia; mitchell.torok@unsw.edu.au (M.T.); melvin.chan@unsw.edu.au (M.C.M.C.); d.sui@unsw.edu.au (D.S.)

**Keywords:** quadrotors, online wind mapping, nonlinear disturbance observer, model predictive contouring control, wind-aware path planning

## Abstract

Quadrotors performing sensing missions near structures such as turbines, towers, and buildings must hold a stable attitude while traversing the structured wind wakes generated by these structures. To maintain trajectory tracking in wind, the vehicle must tilt continuously, and the inner-loop controller must work harder to hold that tilt against the fluctuating flow, raising mean tilt, angular jerk, and command-rate activity. These attitude-domain costs can degrade onboard imagery and gimbal-stabilized sensor data, consuming the actuator authority required to reject further disturbances. Existing work typically treats the two halves of this problem separately: wind is either estimated locally and compensated reactively, or routed around in fields assumed known a priori, and is rarely validated against attitude-domain metrics on hardware. These approaches are most effective when coupled through a single shared representation. A nonlinear disturbance observer estimates wind from the vehicle’s translational dynamics and accumulates it into a spatial map, which simultaneously provides per-stage feedforward compensation to a contouring controller and weights a wind-aware A* planner. On hardware, the estimator matches anemometer ground truth to within 1m/s, and a 2×2 ablation study across three wind configurations shows a reduction of up to 38% in tilt RMS and 28% in its 95th percentile relative to a wind-naive baseline, at the cost of longer paths.

## 1. Introduction

Unmanned aerial vehicles (UAVs) are increasingly deployed in outdoor industrial applications, including renewable infrastructure inspection [1,2], agricultural monitoring [3], and search and rescue [4]. Many of these missions involve sensing tasks, where a mapping payload, an oblique-view camera, or a gimballed instrument must be carried along a prescribed route while the airframe maintains a stable attitude, since excessive body motion blurs imagery, degrades gimbal compensation, and corrupts photogrammetric reconstruction. Such missions are often flown near the structures that generate the most challenging wind conditions, such as the wakes and channeled flow around turbines, towers, and buildings. In these environments, the disturbance is strong, spatially structured, and quasi-steady over the timescale of a flight.

The cost of wind has most often been framed as energy consumption [5], but for sensing missions, a second cost is more immediate. To maintain trajectory tracking in the presence of a steady disturbance, a multirotor must continuously tilt into the wind, and the inner-loop controller must command larger and more frequent corrections to sustain that tilt against fluctuations in the flow, producing measurably higher mean tilt, angular jerk, and command-rate activity. These attitude-domain quantities bound the quality of payload data and correlate with the actuator authority left in reserve to reject further disturbance. Once that reserve is exhausted, tracking fails. Actuator-aware quadrotor MPC has examined related quantities for time-optimal flight at the edge of the actuation envelope [6,7]; the problem here is the complementary one of keeping the vehicle out of that high-effort regime by choosing where it flies as well as how it is controlled.

Since airframe-mounted anemometers are corrupted by propeller wash, wind is not directly measurable on a flying multirotor and is, therefore, inferred from the residual between expected and measured behavior; indirect estimation methods of this kind are surveyed in [8]. Extended-state and Kalman-filter observers couple this residual with a predictive controller [9,10,11]; dynamic-model estimators infer wind from modeled versus measured forces [12]; motion-based estimators recover it from inertial measurements [13]; data-driven estimators regress it directly from flight telemetry [14]; adaptive and learned schemes add an online wind-compensation term or adapt to wind-induced residual dynamics in flight [15,16,17] and incremental nonlinear dynamic inversion rejects disturbance without an explicit estimate [18]. In all of these, the estimate is used reactively: applied at the current position and discarded, never accumulated into a representation of how the disturbance varies over the workspace. A vehicle re-entering a region cannot anticipate the wind there, and a planner has no disturbance information to act on; validation is reported almost exclusively in the position-tracking and energy domains, rarely in the attitude domain.

A complementary body of work treats wind as a spatial field, and plans routes through it: graph search over wind-dependent edge weights [19,20], Markov decision processes for time-optimal routing [21], robust planning under stochastic wind [22], and offline-learned policies [23]. Three assumptions recur: the wind field is known a priori from observatories or simulation rather than being sensed; validation is almost entirely performed in simulation and the objective is a scalar trip cost such as time, energy, or risk, rather than the attitude effort the route imposes on the controller. The two pieces of literature are thus disconnected: estimation produces a local estimate that it does not retain, and planning consumes a global field that it does not measure.

**Figure 1:**
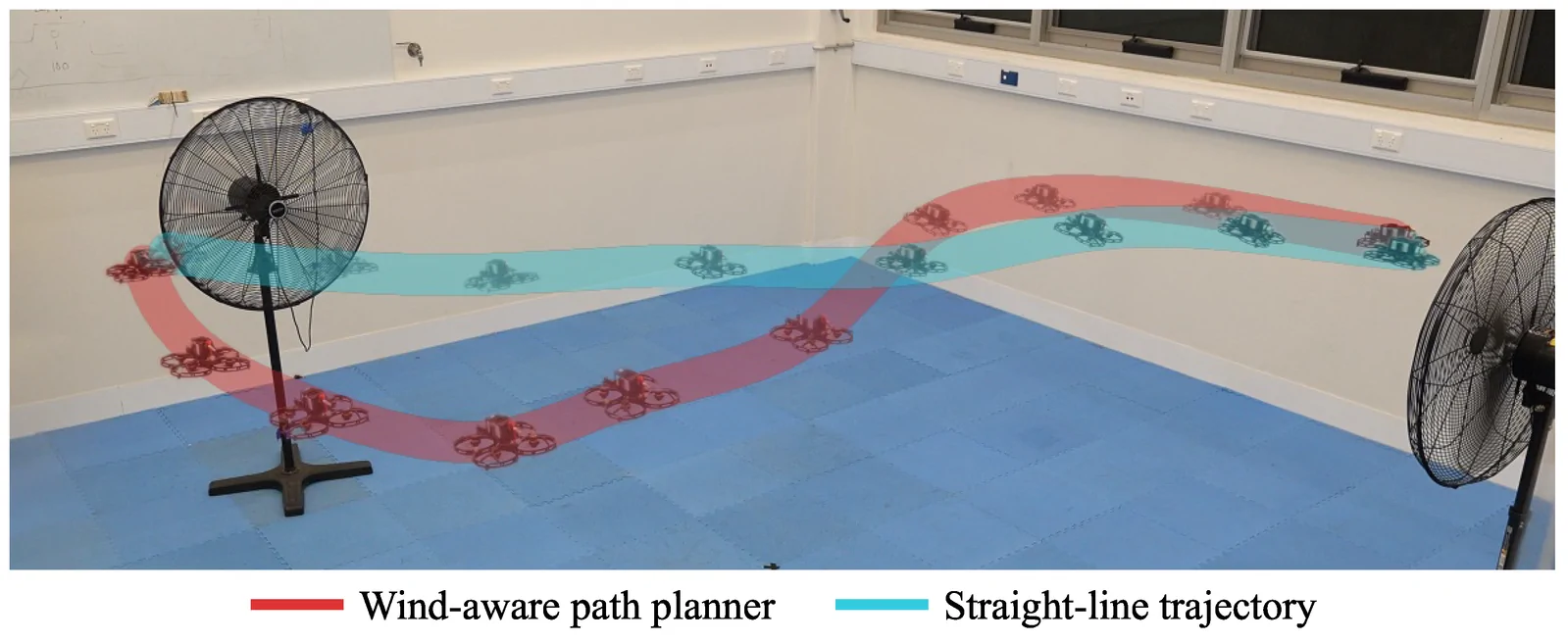
Trajectory comparison under Configuration 2. Multi-exposure composite of the quadrotor flown between the same start and goal waypoints, once using the wind-aware path planner (red) and once with a straight-line trajectory (cyan). Each ghost is the drone segmented from a single video frame and blended onto the scene, while the wide translucent band traces the flight path.

This work closes that gap with a system in which the wind field is (i) estimated online from the vehicle’s translational dynamics, (ii) accumulated into a workspace-level map shared between per-stage feedforward at the controller and edge weighting at the planner, and (iii) validated on hardware against attitude-domain metrics rather than tracking error or energy alone. The planner is driven by the attitude deflection each route would demand of the controller, so it routes away from strong disturbance while the controller anticipates the residual wind. Figure 1 previews the effect, with the wind-aware planner routing around the fan jet while a straight-line baseline maintains trajectory tracking at the cost of directly counteracting the disturbance. The closest prior points of contact are [12], which estimates wind from multirotor dynamics on real hardware but without spatial memory or coupling to a planner, and [24], which accumulates sparse in-flight measurements into a dense wind field over known terrain but does not couple the prediction to the planning or control of the vehicle that gathers them. The target regime is structured, quasi-steady wind fields, the wakes and channelled flows around buildings and infrastructure, whose topology persists long enough to be learned over a few traversals. Stochastic gusts are outside the scope. Relative to the wind-estimation literature, estimates are retained rather than applied at the current position and discarded, so the disturbance learned on one traversal is available to every subsequent query. Relative to wind-aware planning, the field is measured by the vehicle during the mission rather than assumed known a priori, and the objective is the attitude effort a route imposes on the controller rather than a scalar trip cost. Relative to predictive control with wind compensation, where the current estimate is typically held constant over the prediction horizon, the feedforward here is evaluated from the map at each stage, so the controller anticipates wind the vehicle has not yet reached.

The contributions of this work are as follows:

- A nonlinear disturbance observer that estimates wind from the vehicle’s translational dynamics, validated against a static anemometer at known positions across the test volume. Estimates are accumulated during flight into a workspace-level spatial wind map that extrapolates each observation along the prevailing wind direction.
- Joint exploitation of that map by a model predictive contouring controller, which applies per-stage wind feedforward along its horizon, and a wind-aware A* planner, whose edge weights penalize the predicted attitude deflection required to traverse each cell. The same learned representation thus drives both disturbance rejection and route choice.
- A real-hardware 2×2 ablation of the planner and the wind-map feedforward, replicated across three fan configurations, reporting tilt RMS, tilt 95th percentile, angular jerk RMS, and command-rate RMS in the attitude domain alongside path length and per-leg energy as trade-offs. The integrated system reduces tilt RMS by up to 38% and its 95th percentile by up to 28% relative to a wind-naive baseline, at a cost of approximately 38% longer paths.

## 2. Methodology

**Figure 2:**
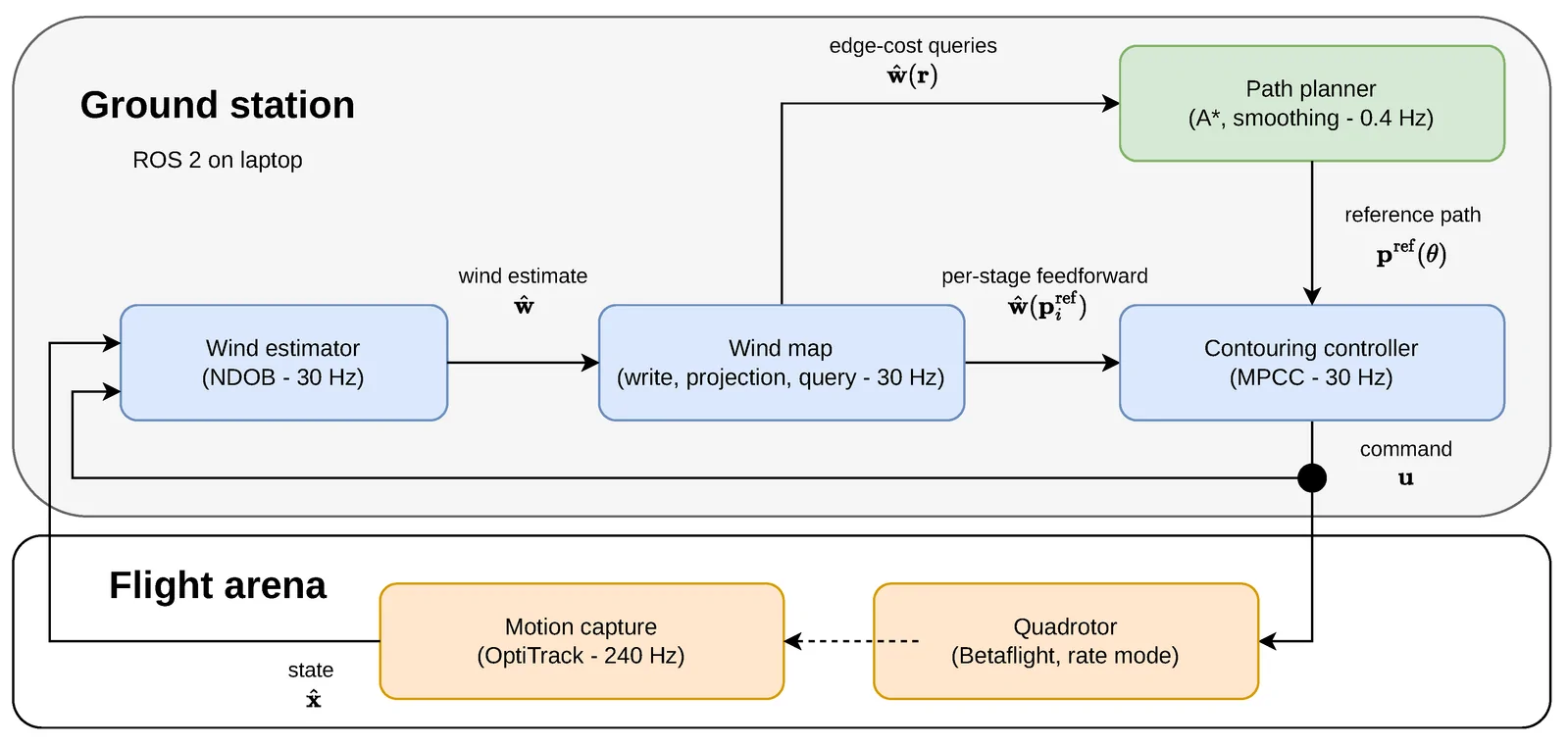
System overview. Motion capture provides the vehicle state to the off-board ground station, where the disturbance observer estimates the wind and accumulates it into the map of Section 2.2. The map is consumed twice, weighting the edges of the A* planner and providing per-stage feedforward along the MPCC horizon, while the planner supplies the reference path the controller tracks and the controller sends body-rate commands to the vehicle over the radio link. The planner is re-run periodically (0.4 Hz, 1.0 Hz in Configuration 1) and at each waypoint arrival. The vehicle state is distributed to all ground-station components, and only primary signal paths are shown.

The proposed system combines a quadrotor dynamics model with wind as an input, an online disturbance observer, a spatial wind map accumulating its estimates, and a planner and controller that exploit the map at runtime (Figure 2). The planner and the controller’s wind-map feedforward can each be enabled independently.

### 2.1. Quadrotor Dynamics

The vehicle is modeled as a nonlinear state-space system

(1) $$\dot{x}=f\left(x,u,w\right),$$

with state x, control input u, and exogenous wind disturbance w, each defined below. The state vector is defined as

(2) $$x={\left[\begin{array}{cccc} p & q & v & \omega \end{array}\right]}^{⊤}\in R^{13},$$

where $p\in R^{3}$ is the position in the inertial world frame W, $q\in R^{4}$ is the unit attitude quaternion giving the orientation of the body-fixed frame B with respect to W, $v\in R^{3}$ is the world-frame translational velocity, and $\omega \in R^{3}$ is the body-frame angular velocity. The body frame B is located at the vehicle’s center of mass.

The flight-controller firmware runs in rate mode and receives the normalized input vector

(3) $$u={\left[\begin{array}{cccc} u_{T} & u_{\phi } & u_{\theta } & u_{\psi } \end{array}\right]}^{⊤}\in R^{4},$$

where $u_{T}\in \left[0,1\right]$ is collective throttle and $u_{\phi },u_{\theta },u_{\psi }\in \left[-1,1\right]$ are roll, pitch, and yaw rate commands. The position and attitude evolve through the kinematic relations

(4) $$\dot{p}=v,\;\dot{q}=\frac{1}{2}\;q⊗\left[\begin{array}{c} 0\\ \omega \end{array}\right],$$

with ⊗ the quaternion product. The translational dynamics under a wind field $w\in R^{3}$, expressed in W, are

(5) $$\dot{v}=a_{T}+g-D\left(v-w\right),$$

where $a_{T}=R\left(q\right){\left[0,0,k_{T}u_{T}\right]}^{⊤}$ is the world-frame thrust acceleration, R(q)∈SO(3) is the rotation from B to W, $k_{T}$ is the throttle gain, $g={\left[0,0,-g\right]}^{⊤}$ is gravitational acceleration, and $D=\mathrm{diag}(d_{x},d_{y},d_{z})$ collects the per-axis drag coefficients. Wind is modeled along the lateral axes only, $w={\left[w_{x},w_{y},0\right]}^{⊤}$, as vertical wind is poorly observable from translational acceleration. A linear drag model outperforms a quadratic one for multirotor wind estimation below approximately 10m/s [12].

The angular velocity follows a first-order response to the rate command,

(6) $$\dot{\omega }=\frac{1}{\tau_{r}}\left(r_{\max}\left[\begin{array}{c} u_{\phi }\\ u_{\theta }\\ -u_{\psi } \end{array}\right]-\omega \right),$$

where $r_{\max}$ is the maximum commanded rate and $\tau_{r}$ the rate-loop time constant; the firmware uses a linear rate curve, so commands map proportionally to body rate up to $r_{\max}$. Equations (4)–(6) together define the dynamics f in (1).

### 2.2. Wind Estimation and Mapping

A nonlinear disturbance observer (NDOB) [25] estimates the wind from the residual between measured and modeled accelerations. Evaluating this residual directly would require the acceleration $\dot{v}$, which is not measured, and differentiating the measured velocity would amplify its noise; the NDOB instead maintains an internal state $z\in R^{3}$ whose dynamics contain only measured and modeled quantities. The observer dynamics are

(7) $$\dot{z}=-L\left(z+L\;v+a_{T}+g-Dv\right),$$

where $L=\ell \;I_{3}$ is the observer gain with ℓ>0, and the wind estimate is

(8) $$\hat{w}=D^{-1}\left(z+L\;v\right),$$

integrated via forward Euler at the controller rate. Differentiating (8) under the translational dynamics (5) gives the error dynamics of the estimation error $\tilde{w}=w-\hat{w}$ as $\dot{\tilde{w}}=\dot{w}-\ell \;\tilde{w}$: constant wind is estimated exponentially at a rate set by *ℓ*, and the quasi-steady fields targeted in this work yield a bounded estimation error that decreases with increasing gain [25].

The estimate $\hat{w}$ is local to the quadrotor’s position, so estimates are accumulated into a wind map that the planner and controller can query anywhere. The map discretizes the horizontal plane into a uniform grid of square cells of side Δ, with the vertical axis collapsed to a single layer since the fans blow horizontally and cruise altitude is fixed. The cell containing a point p is denoted c(p), obtained by rounding each horizontal coordinate to the nearest multiple of Δ. Each cell stores a first-in, first-out buffer of at most $N_{c}$ samples, and the estimate held by the cell is the arithmetic mean of that buffer, so fresh observations displace stale ones as the workspace is re-observed. At each control step after an initial observer settling period, the current estimate is passed through a first-order low-pass filter with coefficient α and appended to the buffer of the vehicle’s cell, whatever its magnitude, so calm air dilutes any stale projections.

Since the disturbances of interest are coherent streams along a prevailing direction, sufficiently strong observations are additionally projected along their own axis, encoding a coherent-plume prior in which the source lies upstream. An observation is projected only when $∥\hat{w}∥\geq w_{\min}$, which keeps near-noise estimates from being smeared across the map. Let ${\hat{e}}_{w}=\hat{w}/∥\hat{w}∥$ be the wind direction. The projected samples $s_{m}^{+}$ and $s_{m}^{-}$, written *m* cells downstream and upstream of the observation for m=1,…,K, are the observation scaled by a constant factor per cell,

(9) $$\begin{array}{cccc} s_{m}^{+} & =\beta_{\mathrm{down}}^{\;m}\;\hat{w},\; & \; & \mathrm{appended}\;\mathrm{at}\;c\left(p+m\Delta \;{\hat{e}}_{w}\right),\\ s_{m}^{-} & =\beta_{\mathrm{up}}^{\;m}\;\hat{w},\; & \; & \mathrm{appended}\;\mathrm{at}\;c\left(p-m\Delta \;{\hat{e}}_{w}\right), \end{array}$$

where $\beta_{\mathrm{down}}^{\;m}<1$ attenuates samples with distance downstream and $\beta_{\mathrm{up}}^{\;m}>1$ amplifies them toward the presumed source upstream. Any sample below the floor $w_{\min}$ is discarded rather than appended, so one observation writes at most 2K projected cells, a line of cells *K* long in each axial direction. Projection acts only on raw observations and mapped values are never re-projected, so no cell-to-cell feedback exists.

The wind at a query position $r\in R^{3}$ is the average of the samples held near the query cell. Let N(r) denote the set of samples, direct and projected, currently stored in the buffers of c(r) and its neighboring cells, indexed as ${\hat{w}}_{j}$. The queried wind is

(10) $$\hat{w}\left(r\right)=\frac{1}{\left|N(r)\right|}\sum_{{\hat{w}}_{j}\in N\left(r\right)}{\hat{w}}_{j}.$$

The controller’s query pools the 3×3 cell neighborhood centered on c(r), smoothing the feedforward, while the planner’s per-edge query reads the single cell c(r), preserving the spatial resolution of the edge costs.

### 2.3. Wind-Aware Path Planning

The planner minimizes the predicted attitude deflection along the route, the steady-state tilt the vehicle must hold to track its intended ground velocity against the mapped wind. Penalizing tilt at the planning stage preserves the roll and pitch margins the controller relies on for further disturbance rejection. Each grid cell is a node with edges to its in-grid neighbors, and the wind query (10) is evaluated at each edge. For an edge from cell $r_{1}$ to neighbor $r_{2}$ of length $d=∥r_{2}-r_{1}∥$ and unit direction ${\hat{e}}_{12}=\left(r_{2}-r_{1}\right)/d$, holding the cruise speed $v_{\mathrm{cruise}}$ along ${\hat{e}}_{12}$ against the queried wind $\hat{w}\left(r_{1}\right)$ requires the airspeed $v_{\mathrm{air}}=v_{\mathrm{cruise}}\;{\hat{e}}_{12}-\hat{w}\left(r_{1}\right)$, balanced by the steady-state tilt

(11) $$\phi \left(r_{1},r_{2}\right)=\arctan\;\left(\frac{k_{\mathrm{drag}}\;∥v_{\mathrm{air}}∥}{g}\right),$$

where $k_{\mathrm{drag}}=d_{x}=d_{y}$ is the lateral drag coefficient, identified as equal on both lateral axes for this platform. The edge cost combines this tilt with a direction-agnostic wind-magnitude term,

(12) $$C\left(r_{1},r_{2}\right)=d\left(1+k_{\phi }\;\phi^{2}+k_{w}\;{∥\hat{w}\left(r_{1}\right)∥}^{2}\right),$$

with weights $k_{\phi },k_{w}\geq 0$. The tilt term captures wind’s directional asymmetry: at a slow cruise reference, headwinds, tailwinds, and crosswinds all enlarge $∥v_{\mathrm{air}}∥$ and hence the required tilt. The magnitude term discourages routes through strong-wind cells where the estimate is least reliable. Since the cost is bounded below by the edge length, straight-line distance is an admissible heuristic and the least-cost path is found with $A^{*}$, then shortcut-smoothed and Chaikin-rounded into the reference path.

### 2.4. Wind-Aware Model Predictive Contouring Control

Unlike standard time-indexed tracking, which counters wind-induced delays to maintain a tight trajectory, Model Predictive Contouring Control (MPCC) decouples physical location from arrival time. Over the prediction horizon, the MPCC computes the sequence of control inputs $u_{i}$ and path-progress speeds $v_{\theta ,i}$ that minimizes the path-tracking, attitude, and control-effort costs subject to the system dynamics and operational constraints; this optimization is formulated below.

The reference path is parameterized by an arc-length progress variable θ, giving a reference position $p^{\mathrm{ref}}\left(\theta \right)$, unit tangent t(θ), and attitude $q^{\mathrm{ref}}\left(\theta \right)$. The quadrotor state (2) is augmented with θ to form the prediction state $x_{i}$, the state predicted at the *i*-th stage of the horizon (i=0,…,N), with $x_{0}={\hat{x}}_{k}$ the current state estimate. Progress evolves through a commanded progress speed $\dot{\theta }=v_{\theta }\geq 0$, which the optimizer sets per stage and is distinct from the fixed cruise speed $v_{\mathrm{cruise}}$ used by the planner. The decision variables are, therefore, the inputs $u_{i}\in R^{4}$ and the progress speeds $v_{\theta ,i}$.

Letting $e_{i}^{\mathrm{pos}}=p_{i}-p_{i}^{\mathrm{ref}}$ be the position deviation, it is resolved into a tangential lag error and a perpendicular contour error,

(13) $$e_{i}^{l}=t_{i}^{⊤}e_{i}^{\mathrm{pos}}-\left(\theta_{i}-\theta_{i}^{0}\right),\;e_{i}^{c}=e_{i}^{\mathrm{pos}}-\left(t_{i}^{⊤}e_{i}^{\mathrm{pos}}\right)\;t_{i},$$

where $\theta_{i}^{0}$ is the progress at which the path is evaluated, held fixed at the value from the previous solver iteration to keep the error dynamics affine. Driving the lag error to zero aligns the reference point with the vehicle’s projection onto the path, so the contour error captures true lateral deviation. The attitude error is $e_{i}^{\mathrm{att}}=\log\left(q_{i}^{\mathrm{ref}}⊗q_{i}^{-1}\right)\in R^{3}$.

At each control step *k*, a warm-started real-time iteration solves

(14) $$\begin{array}{cc} \min_{\left\{u_{i},\;v_{\theta ,i}\right\}} & \sum_{i=0}^{N}\left(∥e_{i}^{c}{∥}_{Q_{c}}^{2}+w_{l}\;{\left(e_{i}^{l}\right)}^{2}+{∥e_{i}^{\mathrm{att}}∥}_{Q_{a}}^{2}\right)\\ \; & +\sum_{i=0}^{N-1}\left(∥u_{i}-u^{\mathrm{ref}}{∥}_{R}^{2}+{∥\Delta u_{i}∥}_{R_{\Delta }}^{2}-w_{\theta }\;v_{\theta ,i}\right)\\ s.t. & x_{0}={\hat{x}}_{k},\\ \; & x_{i+1}=f_{\mathrm{RK}4}\;\left(x_{i},\;u_{i},\;v_{\theta ,i},\;\hat{w}(p_{i}^{\mathrm{ref}}),\;\delta t\right),\\ \; & u_{\min}\leq u_{i}\leq u_{\max},\;\Delta u_{\min}\leq \Delta u_{i}\leq \Delta u_{\max},\\ \; & 0\leq v_{\theta ,i}\leq v_{\theta }^{\max},\;q_{x,i}^{2}+q_{y,i}^{2}\leq \frac{1}{2}(1-\cos\phi_{\max}), \end{array}$$

where *N* is the horizon length, ${∥\cdot ∥}_{W}^{2}$ is the squared norm weighted by a positive-definite matrix *W*, and $f_{\mathrm{RK}4}$ is the fourth-order Runge–Kutta discretization of the dynamics (4)–(6), with the per-stage wind $\hat{w}\left(p_{i}^{\mathrm{ref}}\right)$ entering as a spatially-varying feedforward term that lets the optimizer anticipate wind variation along the horizon. The weights $Q_{c},Q_{a}\in R^{3\times 3}$ penalize contour and attitude error, $R,R_{\Delta }\in R^{4\times 4}$ penalize deviation from the hover input $u^{\mathrm{ref}}$ and the input rate $\Delta u_{i}=u_{i}-u_{i-1}$ (with $u_{-1}=u_{k-1}$), and the scalars $w_{l}$ and $w_{\theta }$ penalize lag and reward forward progress. The bounds give the actuator and progress-speed limits, and the final constraint softly limits the tilt from hover to $\phi_{\max}$, with $q_{x,i},q_{y,i}$ the vector components of $q_{i}$. Only the first input $u_{0}$ is applied before the problem is resolved at the next step.

## 3. Experiments

### 3.1. Experimental Setup

**Figure 3:**
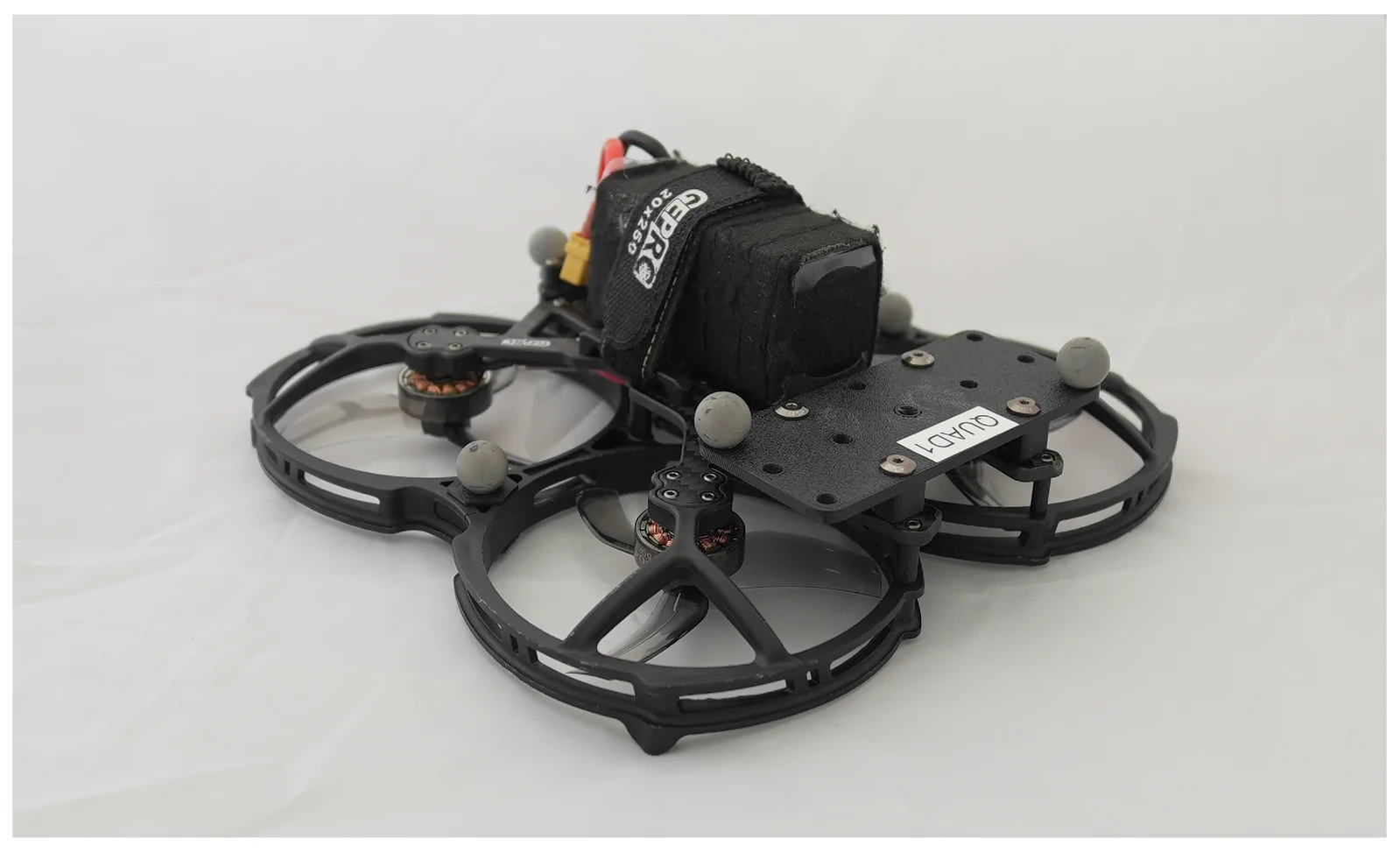
The experimental platform, a 3.5 in ducted cinewhoop quadrotor. Reflective markers on the frame provide the motion-capture pose used for state feedback and wind-map indexing.

Real-world experiments are conducted in a $6\times 6\times 5\;m^{3}$ indoor flight arena instrumented with ten OptiTrack PrimeX 13 motion-capture cameras. The quadrotor 6-DoF pose is extracted by Motive 2.0 and streamed to the ground station, where it is used both as state feedback for the MPCC and as the position input to the wind map. The platform is a cinewhoop-style quadrotor (Figure 3) running Betaflight in rate mode with linear rate curves; its physical parameters are summarized in Table 1. Control runs off-board on a laptop (Intel Core i9-13900H, 32 GB RAM, Ubuntu 22.04, ROS 2), with commands sent over an ExpressLRS link and battery voltage and current returned as telemetry. Each trial used a freshly charged 6S battery at 4.0V per cell (24.0V total) to keep the starting voltage consistent across conditions. Wind is generated by pedestal fans rated up to 6m/s; the number, placement, and orientation of the fans are specified for each experiment. All reported flights are conducted on a fixed-altitude horizontal plane. In preliminary 3D trials, the planner routed over or under the fans, bypassing the disturbance and making the ablation study uninformative; restricting the motion to a plane forces non-trivial interaction with the fan wake and ensures genuine disturbance.

The MPCC runs at a fixed control rate over a 2s receding horizon. The wind map is maintained on a uniform grid, with queries pooling the adjacent-cell neighborhood under 2D operation. Platform model parameters are identified offline; these and the controller, estimator, map, and planner settings are summarized in Table 2. The planner commands a fixed cruise airspeed, and a leg terminates once the vehicle is within one grid cell of its target waypoint. The cruise speed is low, representative of sensing missions and a practical constraint of the indoor arena: at higher speeds the vehicle would traverse the fan jet too briefly to accumulate meaningful tilt. The projection’s downstream-decay and upstream-growth rates were fit to anemometer measurements of fan wind speed at multiple axial distances, and were held fixed across all three configurations. Two experiments evaluate the system: wind-estimation accuracy against anemometer ground truth, and a 2×2 ablation of the planner and wind-map feedforward.

### 3.2. Wind Estimator Validation

**Figure 4:**
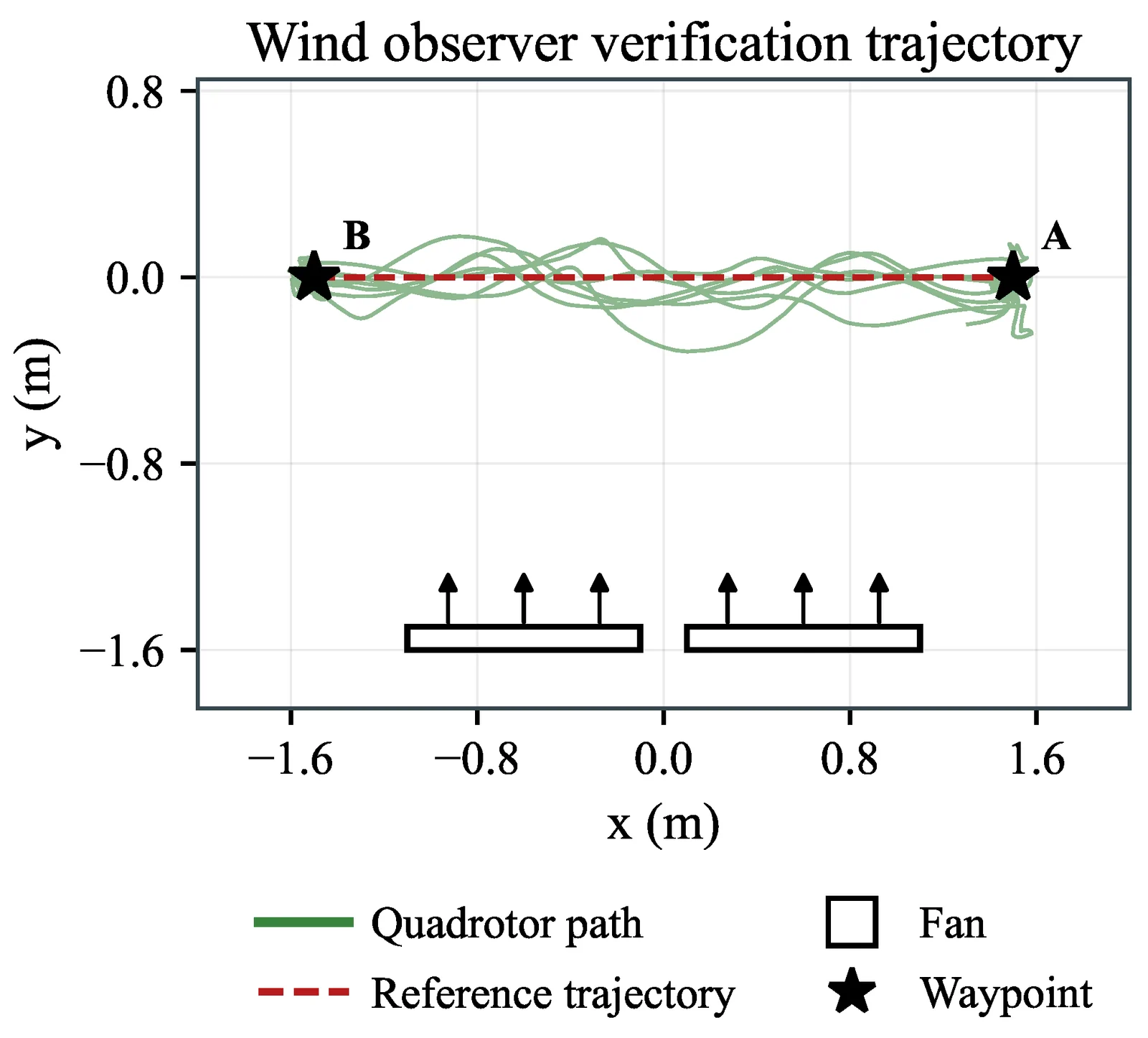
Quadrotor flight path during wind-observer verification. Consecutive A↔B waypoint traversals (green) are overlaid on the straight-line reference path (red dashed). Take-off and landing transits are removed. The hollow rectangles mark the two pedestal fans, with arrows indicating the fan blow direction.

The estimator is validated as the quadrotor flies repeatedly between A=(1.5,0,1.25)m and B=(−1.5,0,1.25)m, crossing a stream from two pedestal fans oriented perpendicular to the flight path as shown in Figure 4, with freestream speeds of 0–5m/s at altitude. NDOB estimates are compared against a tripod-mounted anemometer used as ground truth, positioned at a height of 1.25m, and sampled at 0.25m intervals, recording minimum and maximum wind speeds over a 20s window at each location. Three quadrotor wind estimation trials were conducted, each consisting of six round trips from *A* to *B* and back.

**Figure 5:**
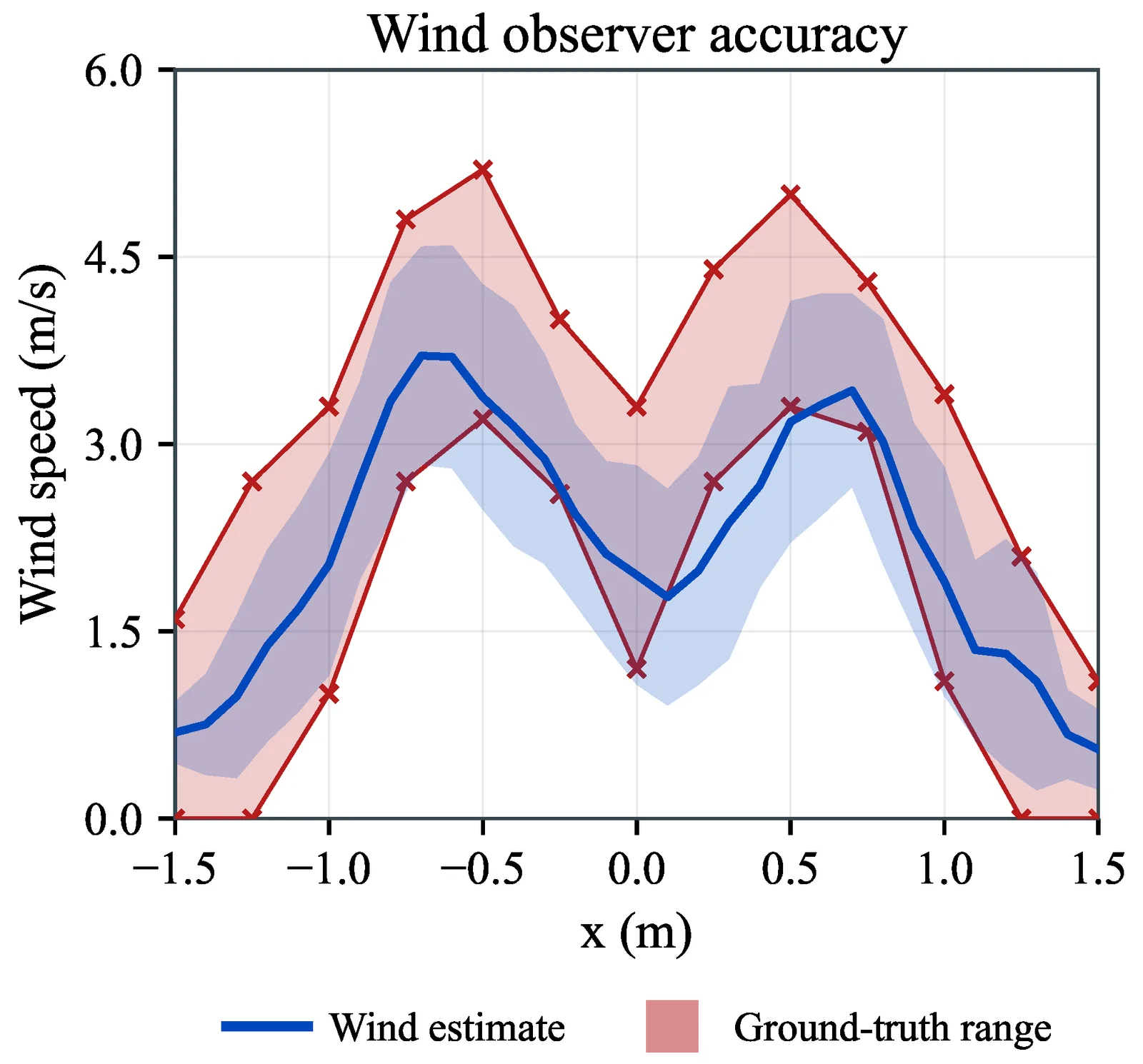
Wind-observer accuracy. The pooled NDOB estimate across three trials (blue, mean ± 1 SD) is overlaid on the anemometer-measured ground-truth envelope (red). The estimate tracks the bimodal fan-jet profile and remains within the measurement uncertainty over the full cage width x∈[−1.5,1.5] m.

Wind estimates are compared against the ground-truth envelope in Figure 5, with per-trial statistics reported in Table 3. The estimator achieves a mean bias of +0.003m/s and an RMSE of 0.72m/s across the three trials. The standard deviation is comparable to that of the dynamic-model estimator of [12] (1.08m/s); however, a direct comparison is not made, as their results were obtained on a different platform under different conditions. With an average error below 1m/s, the estimator is sufficient to populate the map and feed the planner and MPCC.

### 3.3. Path Planner Evaluation

A 2×2 ablation toggles the planner (wind-aware route choice) and the per-stage wind feedforward at the controller, giving four conditions per fan and waypoint configuration (Table 4): Baseline (straight-line path, wind-naive controller); Planner only (planner active but with no map, i.e., still-air assumption; controller wind-naive); Map only (per-stage feedforward on, straight-line path) and Planner + Map (full system, map driving both edge weights and feedforward). These four conditions isolate the two dominant strategies in the literature, reactive disturbance feedforward (Map only) and pre-planned routing over a wind field (Planner only), against the fully-coupled system (Planner + Map). Table 4 reports the cruise-leg metrics for all four conditions, with the best value per metric per configuration set in bold.

The three configurations differ in the geometric relationship between the reference path and the fan jets. In Configuration 1, the fans face the vehicle along most of the path, producing a sustained headwind, the worst case for actuator effort. In Configuration 2, fans on opposing sides of the workspace make the reference path weave between them, producing alternating side-on disturbances. In Configuration 3, the fans are oriented perpendicular to the path, producing pure crosswind with no headwind component. The same fans and freestream speeds are used throughout; only placement and orientation differ. As the primary test, Configuration 1 uses five trials per condition; Configurations 2 and 3 are smaller-scale replications at three trials each, giving $n_{\mathrm{legs}}=55$ and 27 pooled cruise legs, respectively, (takeoff and landing transits excluded) and are reported as indicative.

**Figure 6:**
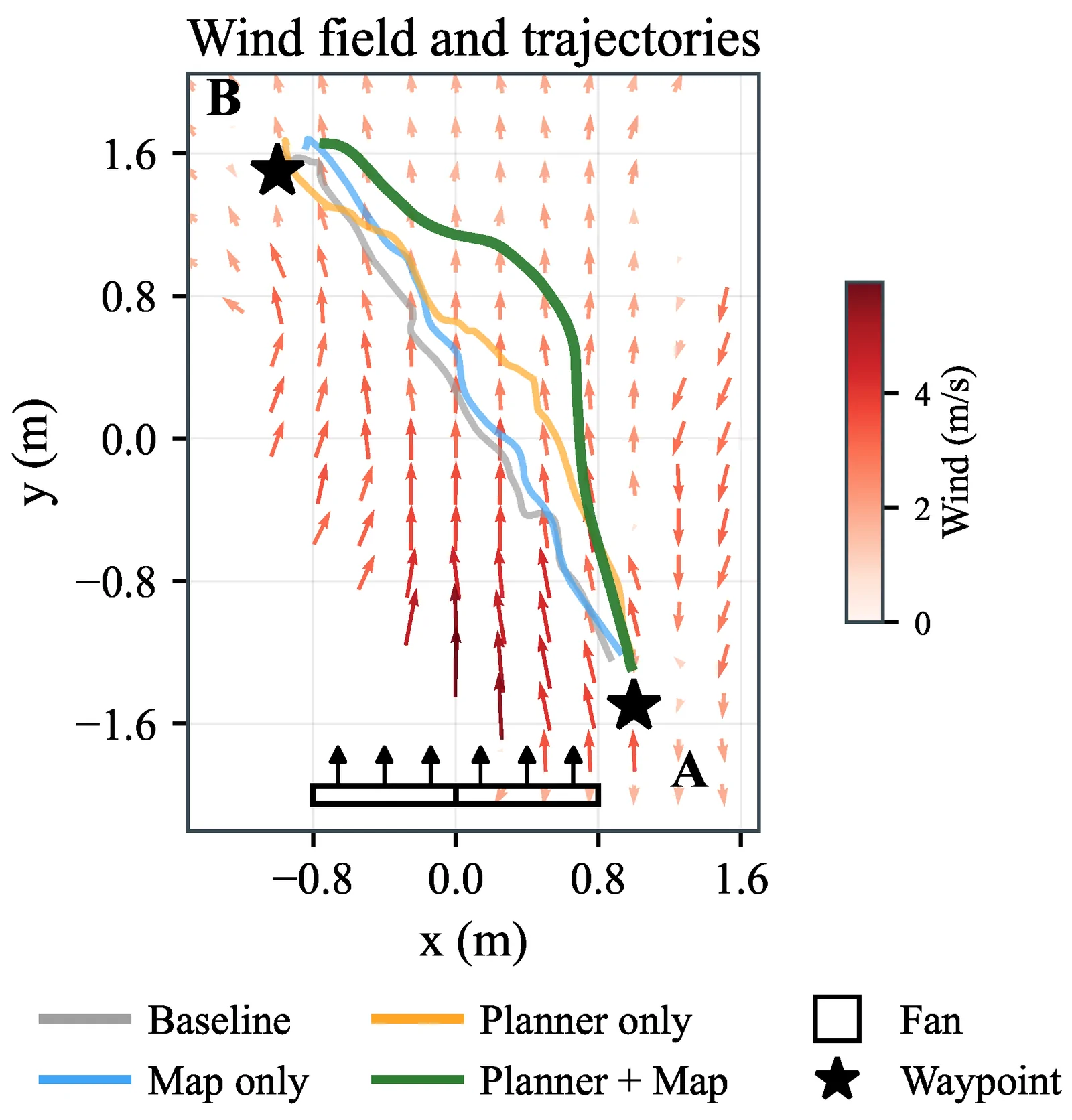
Ablation study: recovered wind field with one representative trajectory per condition. The colored arrows show the NDOB wind estimate averaged across the sampled runs, with color indicating magnitude, and the arrows above the fans indicate the blow direction. Stars mark the waypoints *A* and *B* between which the quadrotor navigates. The Baseline (grey) and Map-only (blue) trajectories pass through the high-wind plume between the fans, while the Planner-only (orange) and Planner + Map (green) routes detour into lower-wind regions.

**Figure 7:**
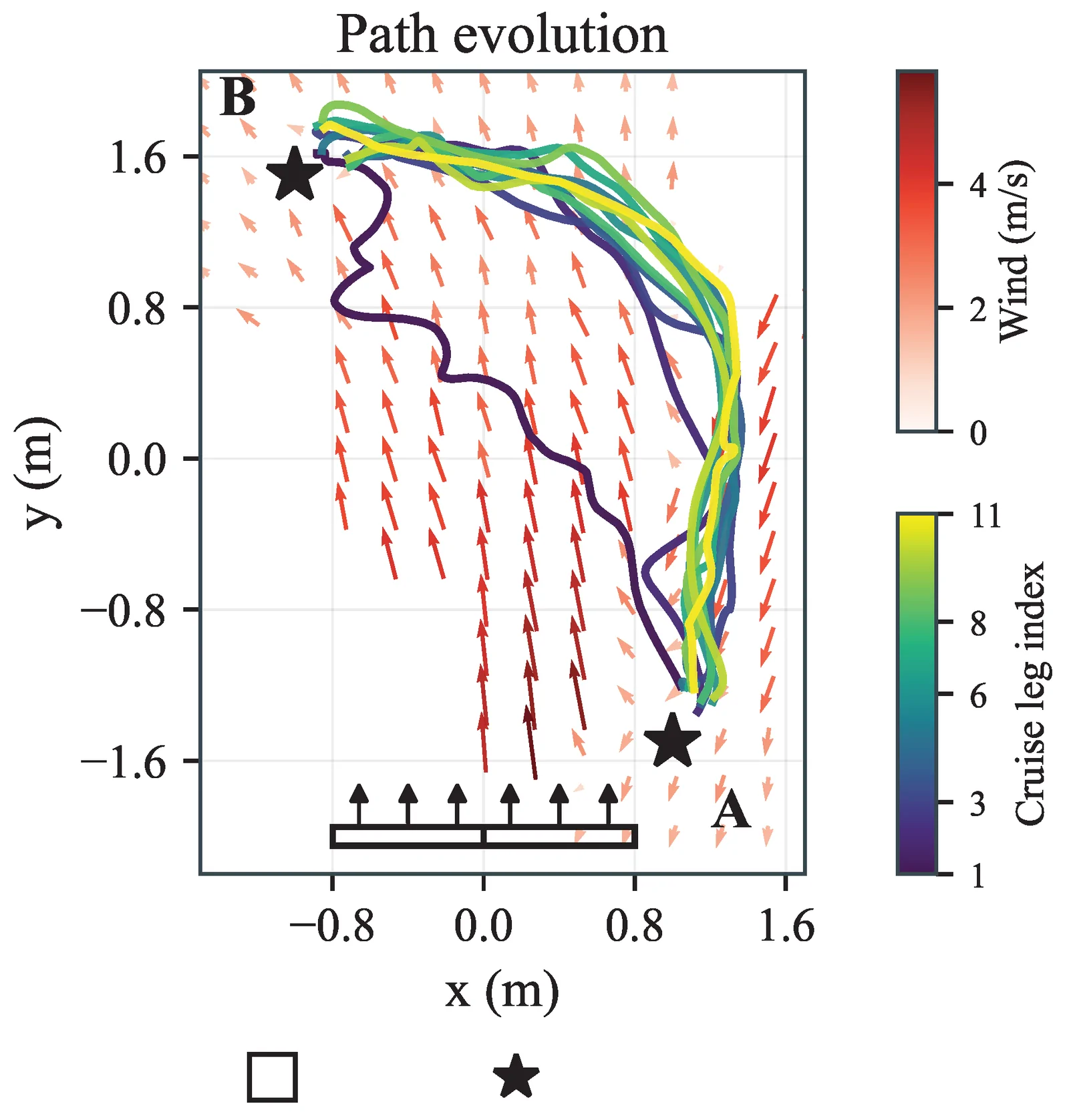
Online path adaptation of the Planner + Map system within a single flight (Configuration 1). Cruise legs between waypoints *A* and *B* are colored by index (1, dark purple, to 11, yellow), over the trial’s end-of-flight NDOB wind map, shown as colored arrows with color indicating magnitude, including the upstream and downstream projections the planner consumes. The arrows above the fans indicate the blow direction. Leg 1 is flown on an essentially empty map and routes near-straight; once enough samples accumulate (from leg 3 onward), the planner commits to the detour, after which the route stabilizes.

In Configuration 1, Planner + Map reduces tilt RMS from $8.7^{\circ }$ to $5.4^{\circ }$ (−38%), tilt p95 from $13.7^{\circ }$ to $9.8^{\circ }$ (−28%), jerk RMS by 10%, and command-rate RMS from 2.63 to 2.15rad/s (−18%). Mean wind sampled along the cruise legs falls by 41%, confirming the planner routes through lower-wind cells (Figure 6). This routing is learned rather than built in. Starting from an empty map, the first leg is flown near-straight, and a single crossing of the jet is sufficient to capture the plume structure, as each strong observation is projected up- and downstream along the prevailing direction. Across trials, the map was populated within the first one to two legs, with the planner committing to the detour by leg 3 in the trial of Figure 7, after which the map and the route were only slightly refined. This warm-up is governed by coverage rather than flight time, as a disturbance must be traversed once before it can be anticipated or avoided; the projection threshold sets how strong that first crossing must be, and the per-cell cap $N_{c}$ sets the timescale of subsequent refinement and forgetting. The pooled metrics of Table 4 include these warm-up legs, so the reported gains include the cost of learning the map. Neither component alone reproduces the result. Jerk and command-rate are not optimized by the planner cost, yet both fall, confirming a genuine reduction in attitude activity rather than an artifact of optimizing the planner’s own objective.

Configurations 2 and 3 replicate the primary effect on different wind topologies. Configuration 2 reduces tilt RMS from $6.7^{\circ }$ to $5.7^{\circ }$ (−15%) and mean wind by 21%, with tilt p95 and jerk largely unchanged. Configuration 3 shows a stronger replication: tilt RMS falls by 20% ($6.8^{\circ }\to 5.5^{\circ }$), tilt p95 by 23%, and mean wind by 24%, on the configuration with the highest baseline p95 of the three. Command-rate is the one attitude metric that does not consistently improve outside Configuration 1: it worsens in Configuration 2 (0.72→0.91rad/s), where the long detour (7.1 vs 5.2m) costs more steering than the mild disturbance justifies, and is essentially unchanged in Configuration 3 (0.67→0.70rad/s). The direction of effect on tilt and wind exposure matches Configuration 1 across both replications, indicating the benefit scales with disturbance severity.

These gains trade off against route cost. Cruise path length grows by 25–62% across the three configurations, and the energy per A↔B round trip rises with it. The spread on the energy metric reflects transit-time variation rather than electrical variation, as the discharge rate was nearly constant across all legs and conditions (1.8±0.1 mAh/s). The widest cell, Planner only in Configuration 1, is dominated by a single flight in which still-air planning stalled against the sustained headwind, with leg durations growing from 30 s to 68 s as successive replans lengthened the path while pack voltage sagged. The flight is retained in the statistics as a genuine failure mode of wind-unaware planning under sustained flow, one that the wind-map conditions did not exhibit. Reporting these alongside the gains makes the operating point explicit. The system buys attitude smoothness with longer routes and modest extra battery draw. The linear, attitude-independent drag model bounds the fidelity of the feedforward, but unmodeled effects such as the change in frontal area with tilt are absorbed into the lumped estimate $\hat{w}$, and since the controller reapplies the drag matrix the observer inverts, the error should largely cancel when samples are reused at similar flight conditions, as the fixed cruise speed maintains here. Within the tilt range flown, the jet crossings were completed without loss of tracking, and the attitude dependence would grow at the speeds and tilts of outdoor flight. Taken across all three configurations, the within-configuration 2×2 ablation and the cross-configuration replication on tilt RMS and wind exposure attribute the improvement to the integrated planner-and-map architecture rather than either component alone.

## 4. Conclusions and Future Work

This paper presents a real-world quadrotor system for waypoint navigation under spatially varying wind, integrating an online disturbance observer, a spatial wind map, a wind-feedforward MPCC controller, and a wind-aware A* planner that queries the same map. On a cinewhoop quadrotor under fan-generated wind, the observer recovered the field to within 1m/s of anemometer ground truth, and a 2×2 ablation across three configurations reduced tilt RMS by up to 38% and its 95th percentile by 28% relative to a wind-naive baseline, at the cost of longer routes. Neither component alone reproduced the combined effect, supporting the central claim that estimation, mapping, and planning are most effective when coupled through a single shared representation.

Several extensions remain. Outdoor deployment should amplify the planner’s benefit in a stronger, more varied flow. A full 3D study would let the planner trade vertical against horizontal detours and yield a volumetric wind map useful beyond control, though densely populating it from few traversals and scaling the search remains open. The map is also only as current as its coverage. A disturbance must be traversed once before it can be anticipated, and a change in an already-mapped region is incorporated only on re-observation, so the planner and feedforward continue to act on outdated estimates in regions the vehicle does not re-visit. The fans were held steady in all trials, and adaptation to mid-flight changes was not tested. Finally, the linear, attitude-independent drag model is coarse; an attitude-conditioned or learned aerodynamic model would propagate into more accurate maps and feedforward.

## Figures and Tables

**Table 1:** Physical parameters of the experimental platform.

Parameter	Value
Frame	CineLog35 V2 cinewhoop frame
All-up mass (incl. battery)	459g
Motors	2004, 1750 KV
Propellers	3.5in, ducted
Flight controller	F7 45A AIO board
Firmware	Betaflight, rate mode
Command link	ExpressLRS
Battery	6S LiPo, 1350mAh

**Table 2:** Identified model parameters and system settings used in all experiments.

Subsystem	Parameter	Symbol	Value
Model	Throttle gain	$k_{T}$	$24.0\;m/s^{2}$
	Drag coefficients	$d_{x},d_{y},d_{z}$	0.45, 0.45, $0.5\;s^{-1}$
	Rate-loop time constant	$\tau_{r}$	0.12s
	Maximum commanded rate	$r_{\max}$	100deg/s
MPCC	Control rate	–	30Hz
	Horizon length	*N*	20 nodes
	Node spacing	δt	0.1s
Estimator	NDOB gain	*ℓ*	5.0 per axis
Wind map	Grid resolution	Δ	0.25m
	Query neighbourhood	–	3×3 cells
	Per-cell sample cap	$N_{c}$	50
	Projection gate	$w_{\min}$	1.0m/s
	Projection extent	*K*	6 cells
	Downstream factor per cell	$\beta_{\mathrm{down}}$	0.841
	Upstream factor per cell	$\beta_{\mathrm{up}}$	1.041
Planner	Cruise airspeed	$v_{\mathrm{cruise}}$	0.5m/s
	Waypoint capture radius	–	0.25m (one grid cell)

**Table 3:** Wind estimation error for Experiment I across three trials: per-axis mean, RMSE, and standard deviation of $\hat{w}-w$ (m/s).

Trial	Bias [m/s]	Std [m/s]	RMSE [m/s]
1	−0.03	0.72	0.72
2	0.08	0.88	0.89
3	−0.04	0.54	0.54
Average	0.003	0.72	0.72

**Table 4:** Cruise-leg metrics across the 2×2 planner and wind-map ablation. Configuration 1 uses n=5 trials per condition (55 cruise legs); Configurations 2 and 3 each use n=3 trials per condition (27 cruise legs). Cells are mean ±1 SD pooled over cruise legs, except the energy row, which is mean ± 1 SD over A↔B round trips (consecutive leg pairs within each trial with a trailing unpaired leg dropped, giving 25 round trips per condition in Configuration 1 and 12 in Configurations 2 and 3). Bold marks the best value per metric per configuration (lower is better; path length and round-trip energy are trade-off rows).

Metric	Baseline (B)	Planner (P)	Map (M)	P + M
Configuration 1				
Tilt RMS [°]	8.7 ± 2.1	7.1 ± 1.5	8.3 ± 2.1	**5.4 ± 1.0**
Tilt p95 [°]	13.7 ± 2.1	11.4 ± 1.6	13.1 ± 2.0	**9.8 ± 1.6**
Jerk RMS [°/s^3^]	2734 ± 159	2607 ± 226	2672 ± 146	**2457 ± 260**
Cmd-rate [rad/s]	2.63 ± 0.20	2.44 ± 0.27	2.52 ± 0.16	**2.15 ± 0.33**
Mean wind [m/s]	2.3 ± 0.6	1.9 ± 0.4	2.1 ± 0.6	**1.4 ± 0.3**
Path [m]	**3.5 ± 0.2**	4.0 ± 1.0	3.6 ± 0.4	4.4 ± 0.7
Energy/round trip [mAh]	**31.0 ± 3.8**	48.9 ± 23.7	32.8 ± 10.1	44.9 ± 8.3
Configuration 2				
Tilt RMS [°]	6.7 ± 0.7	7.1 ± 0.6	6.5 ± 0.8	**5.7 ± 1.1**
Tilt p95 [°]	12.2 ± 1.7	13.0 ± 1.5	11.9 ± 1.6	**11.5 ± 2.6**
Jerk RMS [°/s^3^]	2152 ± 97	2191 ± 167	2151 ± 162	**2109 ± 198**
Cmd-rate [rad/s]	0.72 ± 0.07	**0.70 ± 0.08**	0.75 ± 0.10	0.91 ± 0.17
Mean wind [m/s]	2.3 ± 0.2	2.2 ± 0.3	2.2 ± 0.2	**1.8 ± 0.4**
Path [m]	5.2 ± 1.8	**4.7 ± 0.7**	5.1 ± 1.4	7.1 ± 1.9
Energy/round trip [mAh]	47.2 ± 10.4	**32.9 ± 1.6**	43.6 ± 7.3	49.2 ± 9.0
Configuration 3				
Tilt RMS [°]	6.8 ± 0.8	7.0 ± 1.1	7.3 ± 1.0	**5.5 ± 1.2**
Tilt p95 [°]	14.8 ± 2.0	14.8 ± 1.9	15.4 ± 1.9	**11.5 ± 2.0**
Jerk RMS [°/s^3^]	2028 ± 252	1987 ± 199	1974 ± 167	**1894 ± 200**
Cmd-rate [rad/s]	**0.67 ± 0.10**	0.69 ± 0.11	0.72 ± 0.11	0.70 ± 0.12
Mean wind [m/s]	1.7 ± 0.3	1.6 ± 0.3	1.7 ± 0.3	**1.3 ± 0.3**
Path [m]	2.9 ± 0.8	**2.9 ± 0.5**	3.1 ± 0.5	4.7 ± 1.4
Energy/round trip [mAh]	23.0 ± 2.0	**18.2 ± 1.0**	19.3 ± 0.7	30.1 ± 7.1

## Data Availability

The data presented in this study are available on request from the corresponding author.
